# Homology modeling and molecular dynamics provide structural insights into tospovirus nucleoprotein

**DOI:** 10.1186/s12859-016-1339-4

**Published:** 2016-12-15

**Authors:** Rayane Nunes Lima, Muhammad Faheem, João Alexandre Ribeiro Gonçalves Barbosa, Marcelo Depólo Polêto, Hugo Verli, Fernando Lucas Melo, Renato Oliveira Resende

**Affiliations:** 1 Laboratório de Virologia Vegetal, Departamento de Biologia Celular, Instituto de Ciências Biológicas, Universidade de Brasília, Brasília, DF Brazil; 2 Laboratório de Biofísica, Departamento de Biologia Celular, Instituto de Ciências Biológicas, Universidade de Brasília, Brasília, DF Brazil; 3 Ciências Genômicas e Biotecnologia, Universidade Católica de Brasília, Brasília, DF Brazil; 4 Centro de Biotecnologia, Universidade Federal do Rio Grande do Sul, Porto Alegre, RS Brazil

**Keywords:** Homology modeling, Molecular dynamics, Tospovirus, Nucleoprotein

## Abstract

**Background:**

Tospovirus is a plant-infecting genus within the family Bunyaviridae, which also includes four animal-infecting genera: Hantavirus, Nairovirus, Phlebovirus and Orthobunyavirus. Compared to these members, the structures of *Tospovirus* proteins still are poorly understood. Despite multiple studies have attempted to identify candidate N protein regions involved in RNA binding and protein multimerization for tospovirus using yeast two-hybrid systems (Y2HS) and site-directed mutagenesis, the tospovirus ribonucleocapsids (RNPs) remains largely uncharacterized at the molecular level and the lack of structural information prevents detailed insight into these interactions.

**Results:**

Here we used the nucleoprotein structure of LACV (*La Crosse virus-Orthobunyavirus*) and molecular dynamics simulations to access the structure and dynamics of the nucleoprotein from tospovirus GRSV (*Groundnut ringspot virus*). The resulting model is a monomer composed by a flexible N-terminal and C-terminal arms and a globular domain with a positively charged groove in which RNA is deeply encompassed. This model allowed identifying the candidate amino acids residues involved in RNA interaction and N-N multimerization. Moreover, most residues predicted to be involved in these interactions are highly conserved among tospoviruses.

**Conclusions:**

Crucially, the interaction model proposed here for GRSV N is further corroborated by the all available mutational studies on TSWV (*Tomato spotted wilt virus*) N, so far. Our data will help designing further and more accurate mutational and functional studies of tospovirus N proteins. In addition, the proposed model may shed light on the mechanisms of RNP shaping and could allow the identification of essential amino acid residues as potential targets for tospovirus control strategies.

**Electronic supplementary material:**

The online version of this article (doi:10.1186/s12859-016-1339-4) contains supplementary material, which is available to authorized users.

## Background

*Tospovirus* is a thrips-borne plant-infecting genus within the family *Bunyaviridae*, which also includes four animal-infecting genera: *Hanta/Nairo/Phlebo-* and *Orthobunyavirus* [1]. GRSV (*Groundnut ringspot virus)* is an emerging tospovirus, that has caused severe diseases in distinct vegetable crops in South America and is phylogenetically close to the tospovirus type-species TSWV (*Tomato spotted wilt virus*) [2]. Like all tospoviruses, GRSV contain a trisegmented negative single-stranded RNA (ssRNA) genome that encodes the viral RNA-dependent RNA polymerase (RdRp), two glycoproteins (Gn/Gc), the movement protein (NSm), the RNA silencing suppressor protein (NSs) and the nucleoprotein (N) [3]. N is a multifunctional protein involved in RNA protection, particle assembly, intracellular movement and might play a role in transcription/replication regulation [4–14]. Multiple copies of the N protein form oligomers that interact with the viral RNAs to build ribonucleoprotein complexes (RNPs) that are proposed to be transported via plasmodesmata and are functional templates for RNA replication and transcription [6, 15, 16].

Multiple studies have attempted to identify candidate N protein regions involved in RNA binding and protein multimerization for TSWV using yeast two-hybrid systems (Y2HS) and site-directed mutagenesis [4, 6, 17, 18], but the tospovirus RNPs remains largely uncharacterized at the molecular level and the lack of structural information prevents detailed insight into these interactions. The lack of a reverse genetics system, which is available for other bunyaviruses, has hampered tospovirus research. The N protein crystal structures of related RNA virus families (*Arena*/*Orthomyxo*/*Bunyaviridae*) have been elucidated [8, 19–26] and despite different size and distinct N-folding structures, there are common features and architectural principles by which these proteins form N-N multimers and N-RNA complexes [27]. Therefore, these available structures were used to predict a three-dimensional model for GRSV N (the most important and prevalent tospovirus in Brazil) using homology modeling.

## Results and discussion

### Three-dimensional model of GRSV N and oligomerization

**Fig. 1:**
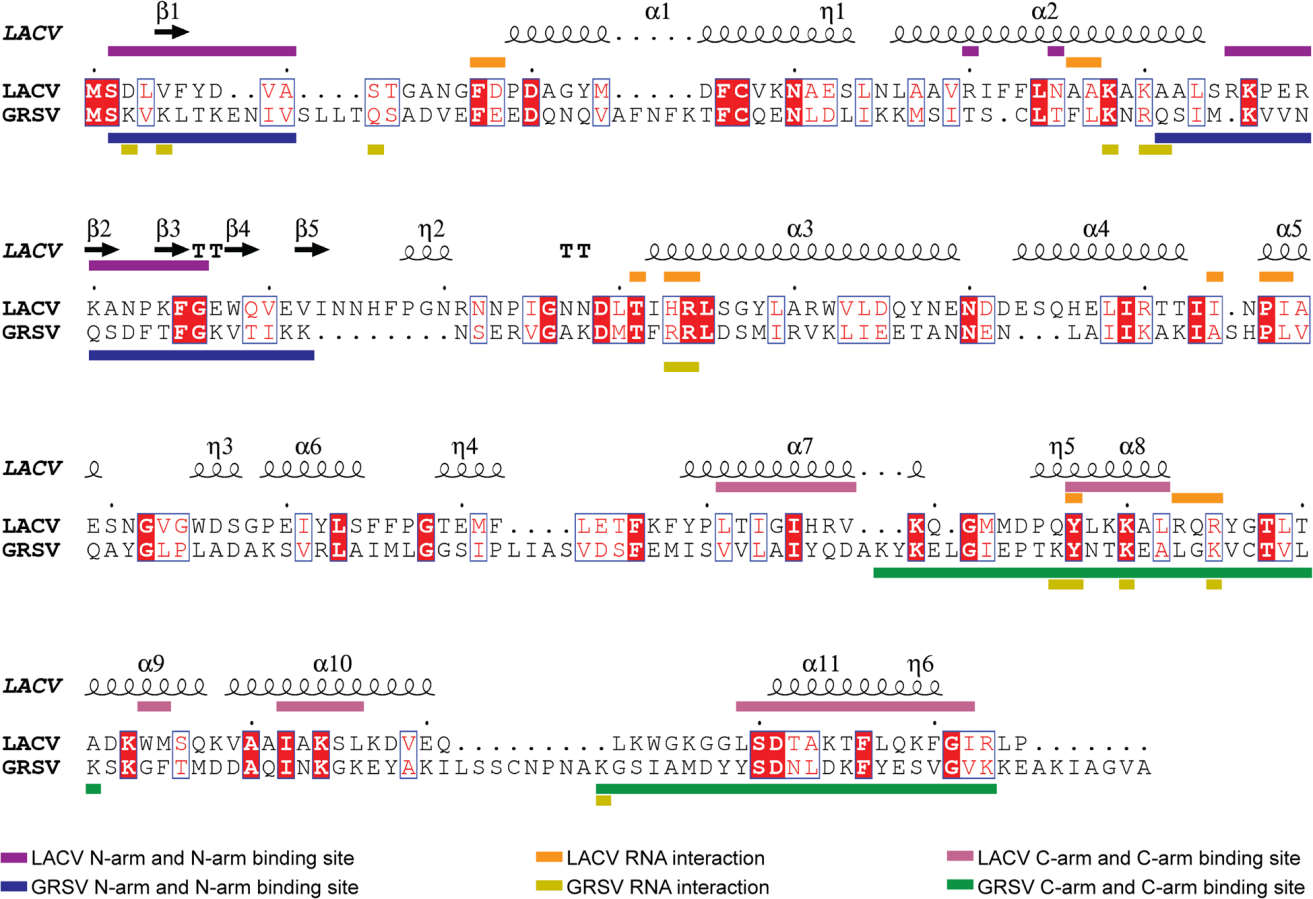
*Groundnut ringspot virus* (GRSV) and *La crosse virus* (LACV) Nucleoproteins sequence alignment. Key residues for GRSV N and LACV N oligomerization and for ssRNA binding are colored as indicated by the colored bars. The secondary structure of LACV N is shown above, and every 10 residues are indicated with a dot (.). Strictly conserved residues are highlighted in red with white letter and highly conserved residues are displayed by red letters. GRSV N GenBank accession number is AF251271 and LACV N UniProt accession code is P04873

**Fig. 2:**
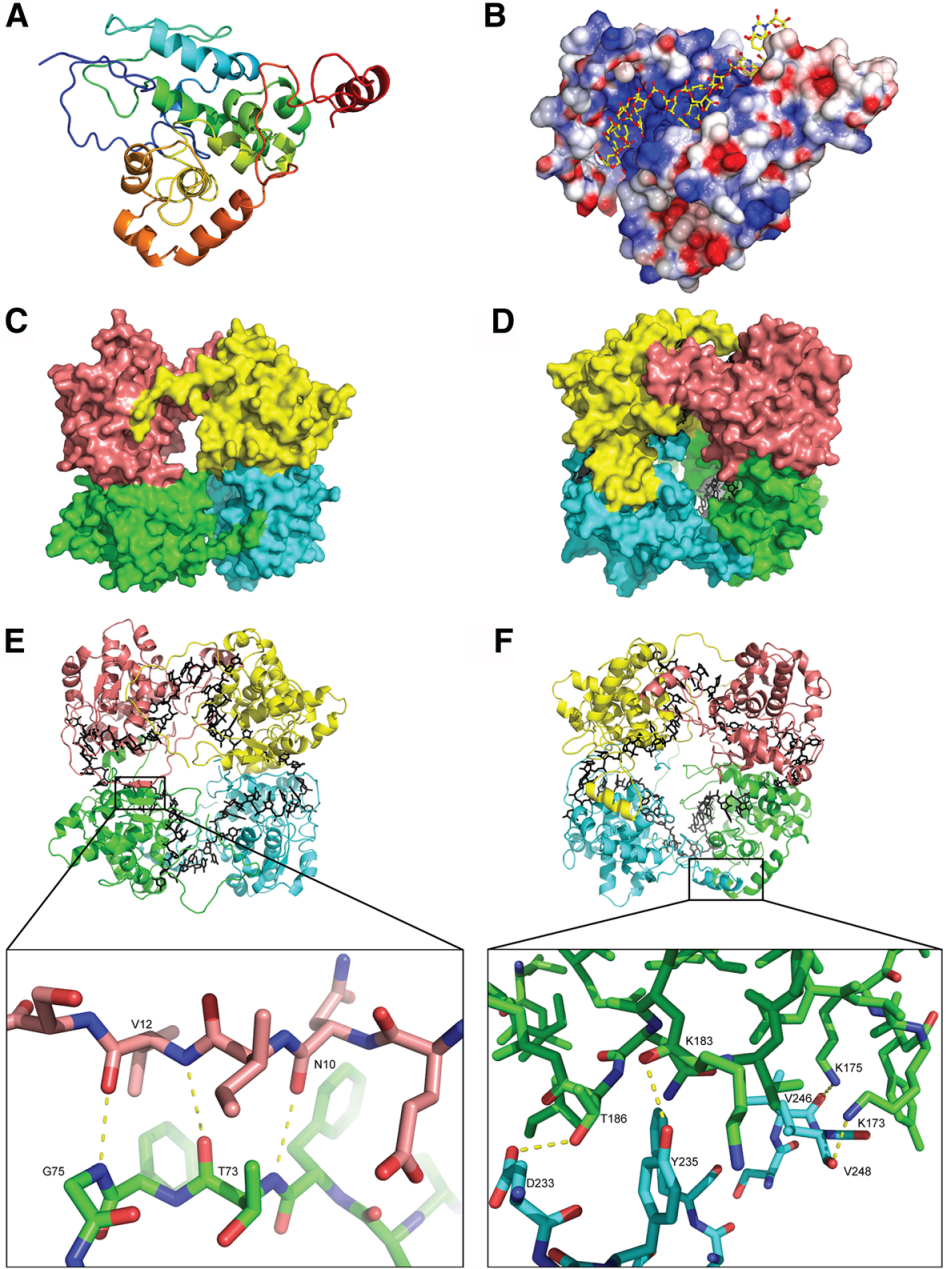
Monomeric and tetrameric structure of the *Groundnut ringspot virus* (GRSV) nucleoprotein (N). **a** Cartoon representation of monomeric GRSV N with rainbow coloring from N- (blue) to C-terminus (red). **b** Electrostatic surface of the GRSV N with a positively charged groove in complex with RNA shown as yellow (carbons) and red (oxygens) sticks. Positive and negative charges are blue and red, respectively. **c** N-terminus interaction surface representation of four GRSV N monomers A, B, C, D shown in color pink, yellow, cyan and green, respectively. **d** C-terminus interaction surface representation of the GRSV N tetrameric ring. The RNA is shown in black sticks deeply bound inside the tetrameric ring. **e** Cartoon representation with the N-arm oligomerization interface showing interacting residues. The N-terminal arm is in pink and the globular region is in green. The intermolecular hydrogen bonds are shown as yellow dotted lines. **f** 180° rotation of Fig. 2e, C-arm oligomerization interface showing interacting residues. The C-terminal arm is in cyan and the globular region is in green

**Fig. 3:**
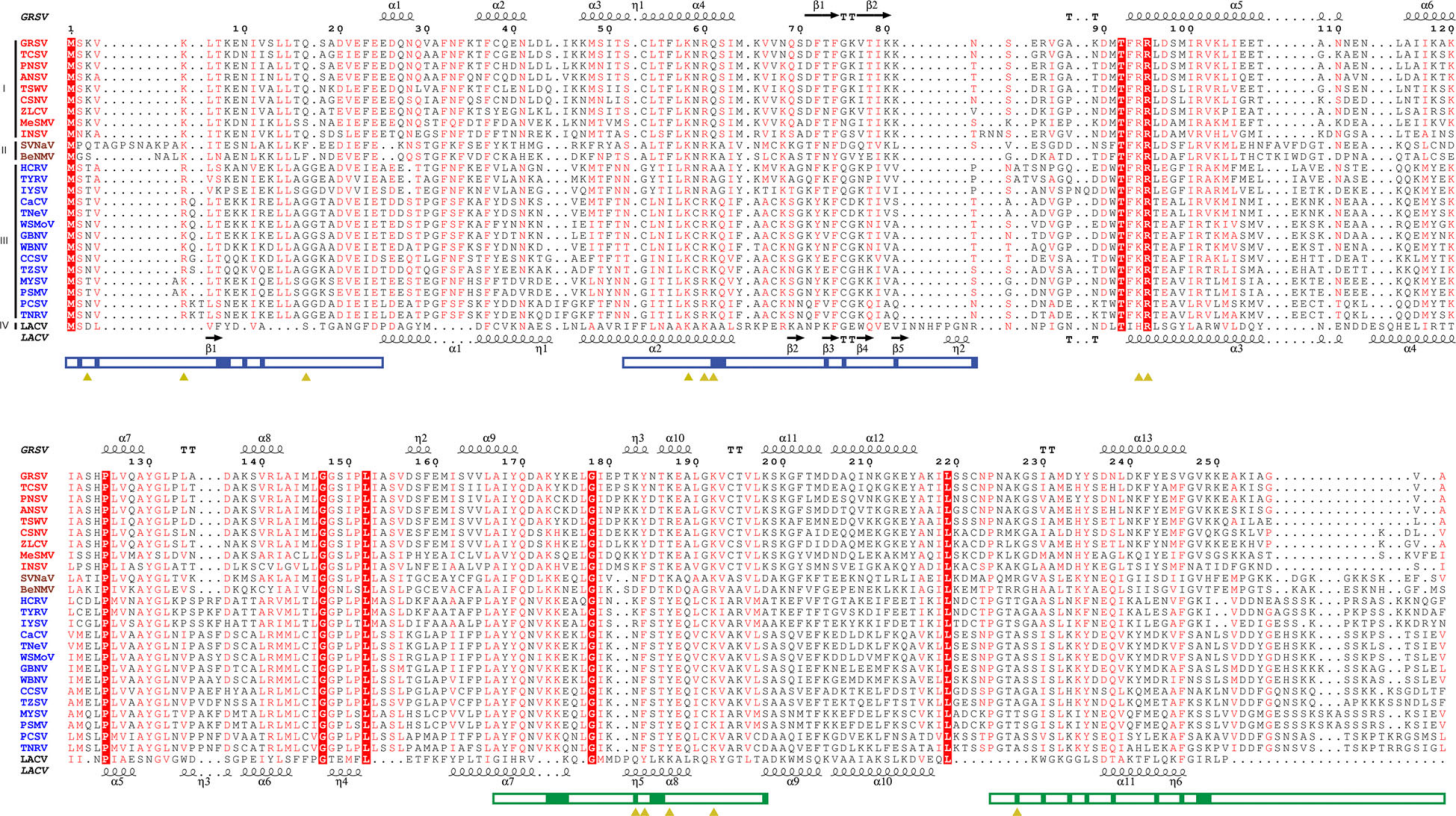
Sequence alignment of representative tospoviruses Nucleoproteins (N). The secondary structure of *Groundnut ringspot virus* (GRSV) is shown above and of *La crosse virus* (LACV) is shown at the bottom. Key residues for GRSV N ssRNA binding are marked with yellow triangles. GRSV N- and C-arms are marked with blue and green boxes respectively, with key residues for oligomerization highlighted. Strictly conserved residues are highlighted in red with white letter and highly conserved residues with red letter. I: *Tospovirus* American clade I; II: *Tospovirus* American clade II; III: *Tospovirus* Eurasian clade; IV: *Orthobunyavirus*. The sequence codes are supplied at the Additional file 2: Table S1

The GRSV N and LACV N have similar protein fold with the predicted GRSV N monomer forming thirteen helical segments and two small beta-sheets (Figs. 1, 2a and e-f). The protein has a globular core domain (26–223 aa) containing a deep positively charged groove with the two chain terminals forming an N-terminus arm (1–25 aa) and a C-terminus arm (224–258 aa) (Fig. 2a-b and Fig. 3). The N- and C-arms extend outwards from the globular core domain and interacts with the globular core domain of neighboring monomers to mediate the multimerization, supporting the “head-to-tail” model proposed by [18]. Amino acids S2-V12 of the N-arm interact with the Q61-N82 of the core domain of one neighboring monomer (Fig. 2c and e) while K227-K249 of the C-arm interact with the K173–K198 of the core domain the other neighboring monomer (Fig. 2d and f). Specific residue-residue interactions have been listed in Table 1 for the two independent interfaces. According to PISA, the intermolecular interactions were mainly hydrogen bonds, but van der Waals and hydrophobic interactions also contribute to hold the monomers together (data not shown). This interaction model is further corroborated by the available mutational studies on TSWV N [4, 17, 18].

**Table 1:** Pairs of interacting residues for GRSV N-N oligomerization

N-arm^a^	N-arm binding site^b^	C-arm^c^	C-arm binding site^b^
S2^d^	S83	A226	K183
V4	N82	S229	K183
T7	Q61	D233	T186
T7	S62	Y235	K183
K8	S62	N238	K198
N10	T73	Y243	N185
V12	T73	V246	K175
V12	G75	V248	K173
		K249	Y174

^a^N-arm amino acids residues of GRSV N

^b^Interacting amino acids residues of GRSV N globular core domain

^c^C-arm amino acids residues of GRSV N

^d^Amino acids residues position in the GRSV N sequence

Actually, the first assay to map functional domains of TSWV N, performing Y2HS and random serial deletions, showed that both the N- (1–39 aa) and C-terminals (233–248 aa) were important for N-N interaction [18], in clear agreement with the structural results presented here. Furthermore, [17] identified three crucial intermonomer binding regions: 42–56, 132–152 and 222–248 which have a clear correspondence with the predicted interaction residues of GRSV N located at N- and C-arms, or buried in the core of the model (Fig. 3). Moreover, amino acids residues located at the regions K103-A119 and L132-V135 are solvent accessible and therefore are able to interact with NSm, glycoproteins, viral polymerase or host proteins [6, 7]. Recently, studies have been performed attempting to identify N-NSm interactions [28, 29] which results are in perfect congruence with the GRSV N protein model. In both cases, the model proposed here represents an efficient tool to assist in planning experiments with mutations and deletion in the N protein.

**Fig. 4:**
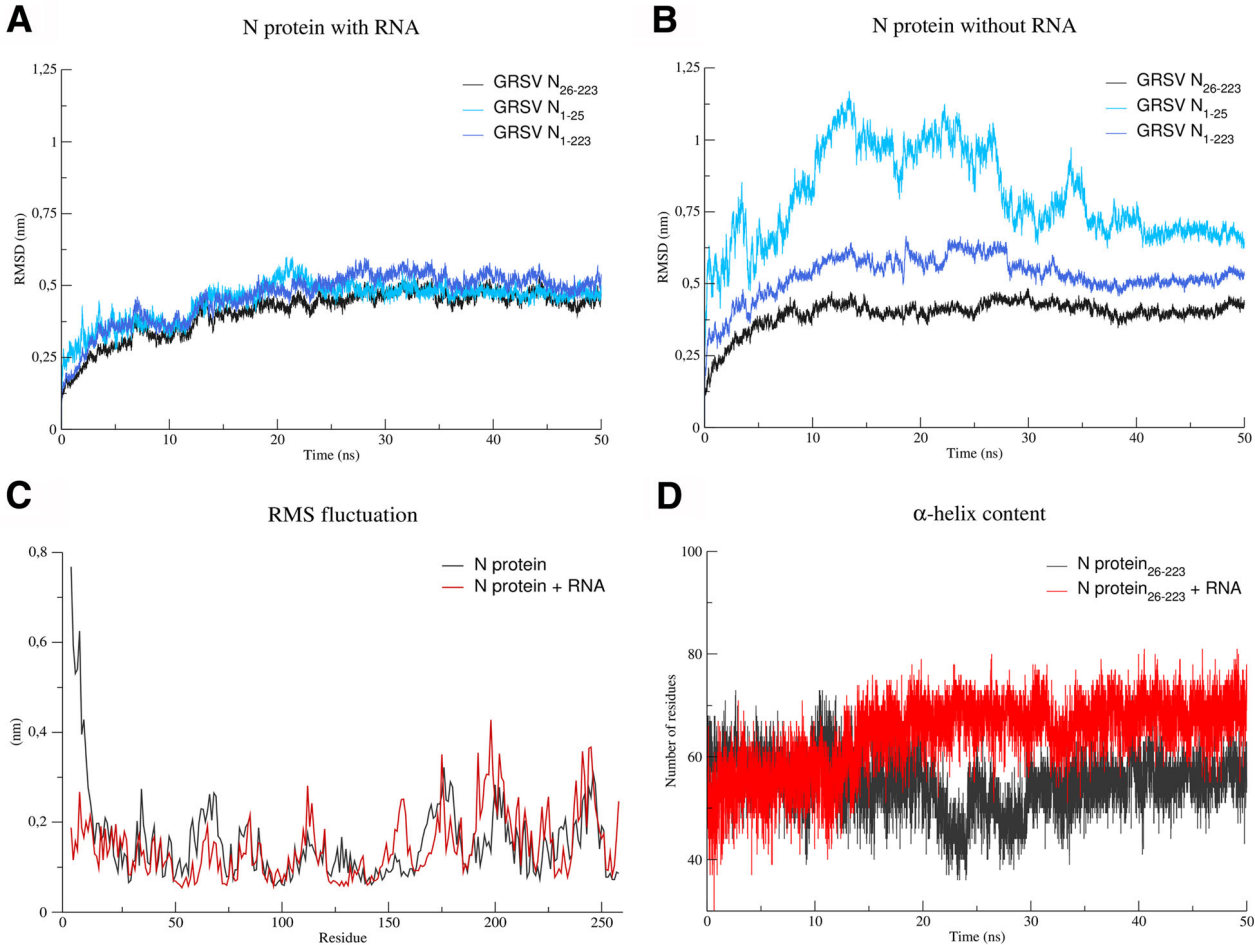
Molecular Dynamics of monomeric Nucleoprotein (N) of *Groundnut ringspot virus* (GRSV). Root Mean Square Deviation (RMSD) calculations for different set of atoms in both presence **a** and absence **b** of RNA. **c** Root Mean Square Fluctuations (RMSF) calculations for the entire N protein in both presence (*red*) and absence (*black*) of RNA. **d** Plot of α-helix content as function of time in both presence (*red*) and absence (*black*) of RNA

In addition, the obtained model for N protein was submitted to molecular dynamics simulations in order to both refine the structure in aqueous solvent [30, 31] and access the protein conformational ensemble, further exploring its structural and functional roles. During the simulation time, the globular core domain did not reveal any loss of secondary structure, increase of radius of gyration or persistent increments on RMSD values, which supports the model quality. It is worthy to mention that RMSF calculations indicate the N-terminal arm (1–25 aa) as a very flexible region (Fig. 4c).

### RNA interaction

According to the GRSV N protein model, the RNA is primarily bound at the central RNA-binding groove (Fig. 2b), and the key residues for this interaction (K3, K5, Q17, K58, R60, Q61, R94, R95, K183, Y184, K187, K192 and K227) are mainly located in this positively charged groove. This positively charged groove is only possible because residues F37, F56, F72, F74, I79, M91, F93 and L96 form a hydrophobic core, which is indispensable to stabilize the protein folding and to correctly orient the RNA interacting residues towards the groove. Importantly, these residues are highly conserved among all tospoviruses (Fig. 3). Note that the N-terminal arm is also involved in RNA binding and shielding RNA from the solvent (Fig. 2c-d). Residues F23, L54, F56, L57 and F93 were observed to modulate the RNA nucleobases dynamics during the performed simulation, while the N-terminal arm seems to play a stabilization role during MD simulations of GRSV N protein (Fig. 4a and b). In addition, the content of alpha-helices in GRSV N protein bound to RNA increased 25 % during the simulation in comparison to the free monomer (Fig. 4d), suggesting that, in the simulated timescale, the monomeric state does not present a lack of conformational stability in detriment of oligomeric states, as observed experimentally for other viruses [32, 33].

Recently, the residues R60, R94, and R95 were confirmed to interact with RNA [33], which also supports our results. RNA is strongly bent at each N-N interface and is largely solvent-inaccessible in the tetramer (Fig. 2d). The dimensions of the groove can accommodate ssRNA and PISA analysis showed that the majority of residue-nucleotide interactions occur with the ribose and the phosphate moieties, suggesting a non-sequence-specific RNA interaction. Indeed, Richmond et al. [4] carried out mutagenesis and gel shift assay studies to identify N regions important for ssRNA binding and demonstrated that the N-RNA complex is highly stable and non-sequence-specific, further supporting these results.

## Conclusions

Taken together, these data will help designing further and more accurate mutational and functional studies of tospovirus N proteins. In addition, the proposed model may shed light on the mechanisms of RNP shaping and could allow the identification of essential amino acid residues as potential targets for tospovirus control strategies.

## Methods

### *In silico* homology modeling and model optimization

A template for modeling the GRSV N protein was searched in expasy SWISS-MODEL server [34] using the amino acid sequence of GRSV N as a reference. Template crystal structures of *Orthobunyavirus* genus were chosen due to their genetic relationship. The LACV (*La Crosse virus-Orthobunyavirus*) N tetrameric crystal structure in complex with ssRNA (PDB ID 4BHH) was selected as the template [20], aligned with GRSV N using T-Coffee server [35] and the resulting alignment was manually improved using BioEdit [36]. Aligned sequences were used with MODELLERv9.10 [37] to develop high quality tetrameric models along with or without RNA.

Optimization of the models was achieved using energy minimization protocols available at Yasara [38] and Chiron [39] servers. Quality of the 3D models were evaluated with ERRAT (version 2.0) [40] and MOL probity [41]. Ramachandran plots for the models were assessed and Ramachandran outlier residues were fixed with COOT [42] and energy minimization. The highest quality model with 90.1 % residues in favored region and 8.4 % in allowed region while 1.5 % outlier at Ramachandran plot was selected after visual inspection (see Additional file 1: Figure S1). The model was subjected to the PISA program [43] for interface analysis at EBI-EMBL server and the retrieved PISA data was analyzed for binding patterns using PyMOL [44].

### Molecular dynamics

Molecular dynamics techniques were applied using GROMACS suite [45] in order to evaluate the stability and consistency of the obtained N protein monomeric model and investigate GRSV N protein-RNA interactions over time. Therefore, N protein model was simulated in the presence and absence of the modeled RNA, in two analytical systems. Amber99SB-ILDN force field [46] was used to generate proper topologies. The models were placed at the center of a dodecahedral box and solvated with TIP3P water model [47]. Counterions were used to neutralize the net charge of the system, and 0.15 M of NaCl was added to the box in order to simulate cellular ionic environment.

After a minimization protocol using steepest descent and conjugate gradient to eliminate possible clashes and bad contacts, NVT ensemble with restraint forces of 1000 kJ/mol was carried for 4 ns at 300 K. Moreover, five subsequent equilibration steps in NPT ensemble were carried out at 1 bar with restraint forces of 800 kJ/mol on heavy atoms, 600 Kcal/(mol x nm) and 400 kJ/mol on mainchain, 200 kJ/mol on backbone and 100 kJ/mol on alpha-carbons, totalizing 13 ns. Finally, production runs with no restraints were carried for 50 ns using an integration step of 2 fs and LINCS algorithm [48]. Also, Particle Mesh Ewald method [49] was applied for Coulombic and Lennard-Jones interactions longer than 1 nm.

## Additional files

Additional file 1: Ramachandran plot analysis of predicted structure of *Groundnut ringspot virus* (GRSV) N protein. The regions covered by light blue lines show most favored regions, while the regions covered by dark blue lines show allowed regions. Other regions of the plot show the disallowed region. The pink dots show the outliers (PNG 87 kb)
Additional file 2: The Genbank acession numbers of the viruses used at this work (TABLEDOCX 19 kb)
