# Supplementary figures and images for: Homology modeling and molecular dynamics provide structural insights into tospovirus nucleoprotein
Source: BMC Bioinformatics. 2016 Dec 15;17(Suppl 18):489. doi: 10.1186/s12859-016-1339-4 (PMC5249003; doi:10.1186/s12859-016-1339-4)

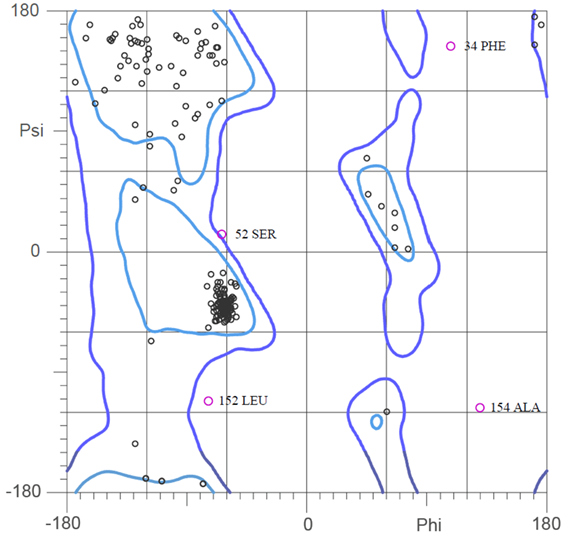

Supplement: Additional file 1: — Ramachandran plot analysis of predicted structure of Groundnut ringspot virus (GRSV) N protein. The regions covered by light blue lines show most favored regions, while the regions covered by dark blue lines show allowed regions. Other regions of the plot show the disallowed region. The pink dots show the outliers (PNG 87 kb) [file 12859_2016_1339_MOESM1_ESM.png]
