# Supplementary material for: Homology modeling and molecular dynamics provide structural insights into tospovirus nucleoprotein
Source: BMC Bioinformatics. 2016 Dec 15;17(Suppl 18):489. doi: 10.1186/s12859-016-1339-4 (PMC5249003; doi:10.1186/s12859-016-1339-4)
Supplement: Additional file 2: — The Genbank acession numbers of the viruses used at this work (TABLEDOCX 19 kb) [file 12859_2016_1339_MOESM2_ESM.tabledocx]

**Additional file 1:** The sequence codes of the viruses used at this work.

| Acronym | Name | Accession number |
| --- | --- | --- |
| ANSV | Alstroemeria necrotic streak virus | GQ478668^a^ |
| BeNMV | Bean necrotic mosaic virus | NC_018071^a^ |
| CaCV | Capsicum chlorosis virus | NC_008301 ^a^ |
| CCSV | Calla lily chlorotic spot virus | AY867502 ^a^ |
| CSNV | Chrysanthemum stem necrosis virus | AF067068 ^a^ |
| GBNV  GRSV | Groundnut bud necrosis virus  *Groundnut ringspot virus* | NC_003619 ^a^  AF251271 ^a^ |
| HCRV | Hippeastrum chlorotic ringspot virus | KC290943 ^a^ |
| INSV | *Impatiens necrotic spot virus* | D00914 ^a^ |
| IYSV | *Iris yellow spot virus* | JQ973066 ^a^ |
| LACV | *La crosse virus*-*Orthobunyavirus* | P04873^b^ |
| MSMV | Melon severe mosaic virus | EU275149 ^a^ |
| MYSV | Melon yellow spot virus | NC_008300 ^a^ |
| PCSV | Pepper chlorotic spot virus | KF383956 ^a^ |
| PNSV | Pepper necrotic spot virus | HE584762 ^a^ |
| PSMV | Physalis severe mottle virus | AB038343 ^a^ |
| SVNaV | Soybean vein necrosis-associated virus | HQ728387 ^a^ |
| TCSV | *Tomato chlorotic spot virus* | JX244197 ^a^ |
| TNeV | Tomato necrosis virus | AY647437 ^a^ |
| TNRV | Tomato necrotic ringspot virus | FJ946835 ^a^ |
| TSWV | Tomato spotted wilt virus | JF808217 ^a^ |
| TYRV | Tomato yellow ring virus | DQ810195 ^a^ |
| TZSV | Tomato zonate spot virus | NC_010489 ^a^ |
| WBNV | *Watermelon bud necrosis virus* | EU249351 ^a^ |
| WSMoV | *Watermelon silver mottle virus* | NC_003843 ^a^ |
| ZLCV | *Zucchini lethal chlorosis virus* | KU641380^a^ |

^a^ GenBank accession number

^b^ UniProt accession number
